# The COVID-19 Sentinel Schools Network of Catalonia (CSSNC) project: Associated factors to prevalence and incidence of SARS-CoV-2 infection in educational settings during the 2020–2021 academic year

**DOI:** 10.1371/journal.pone.0277764

**Published:** 2022-11-17

**Authors:** Fabiana Ganem, Anna Bordas, Cinta Folch, Lucia Alonso, Marcos Montoro-Fernandez, Andreu Colom-Cadena, Ariadna Mas, Jacobo Mendioroz, Laia Asso, Andres Anton, Tomàs Pumarola, Maria Victoria González, Ignacio Blanco, Pere Soler-Palacín, Antoni Soriano-Arandes, Jordi Casabona, on behalf of Sentinel School Network Study Group of Catalonia

**Affiliations:** 1 Centre of Epidemiological Studies on Sexually Transmitted Infections and AIDS of Catalonia (CEEISCAT), Health Department, Government of Catalonia, Badalona, Spain; 2 Departament de Pediatria, d’Obstetrícia i Ginecologia i de Medicina Preventiva i de Salut Publica, Universitat Autònoma de Barcelona, Bellaterra, Spain; 3 Institut d’Investigació Germans Trias i Pujol (IGTP), Badalona, Spain; 4 Spanish Consortium for Research on Epidemiology and Public Health (CIBERESP), Instituto de Salud Carlos III, Madrid, Spain; 5 Direcció Assistencial d’Atenció Primària i Comunitària, Institut Català de la Salut, Barcelona, Catalonia, Spain; 6 Subdirecció general de Vigilància i Resposta a Emergències de l’Agència de Salut Pública de Catalunya, Departament de Salut, Catalonia, Spain; 7 Agència de Salut Pública de Catalunya (ASPCAT), Departament de Salut, Generalitat de Catalunya, Catalonia, Spain; 8 Microbiology Department, Vall d’Hebron Institut de Recerca (VHIR), Vall d’Hebron Hospital Universitari, Vall d’Hebron Barcelona Hospital Campus, Universitat Autònoma de Barcelona, Barcelona, Catalonia, Spain; 9 Microbiology Department, Laboratori Clínic Metropolitana Nord, Hospital Universitari Germans Trias i Pujol, Institut Català de la Salut, Institut D’Investigació en Ciències de La Salut Germans Trias i Pujol (IGTP), Badalona, Catalonia, Spain; 10 Pediatric Infectious Diseases and Immunodeficiencies Unit, Hospital Universitari Vall d’Hebron, Vall d’Hebron Institut de Recerca, Universitat Autònoma de Barcelona, Barcelona, Catalonia, Spain; Zagazig University Faculty of Human Medicine, EGYPT

## Abstract

The Sentinel Schools project was designed to monitor and evaluate the epidemiology of COVID-19 in Catalonia, gathering evidence for health and education policies to inform the development of health protocols and public health interventions to control of SARS-CoV-2 infection in schools. The aim of this study was to estimate the prevalence and incidence of SARS-CoV-2 infections and to identify their determinants among students and staff during February to June in the academic year 2020–2021. We performed two complementary studies, a cross-sectional and a longitudinal component, using a questionnaire to collect nominal data and testing for SARS-CoV-2 detection. We describe the results and perform a univariate and multivariate analysis. The initial crude seroprevalence was 14.8% (95% CI: 13.1–16.5) and 22% (95% CI: 18.3–25.8) for students and staff respectively, and the active infection prevalence was 0.7% (95% CI: 0.3–1) and 1.1% (95% CI: 0.1–2). The overall incidence for persons at risk was 2.73 per 100 person-month and 2.89 and 2.34 per 100 person-month for students and staff, respectively. Socioeconomic, self-reported knowledge, risk perceptions and contact pattern variables were positively associated with the outcome while sanitary measure compliance was negatively associated, the same significance trend was observed in multivariate analysis. In the longitudinal component, epidemiological close contact with SARS-CoV-2 infection was a risk factor for SARS-CoV-2 infection while the highest socioeconomic status level was protective as was compliance with sanitary measures. The small number of active cases detected in these schools suggests a low transmission among children in school and the efficacy of public health measures implemented, at least in the epidemiological scenario of the study period. The major contribution of this study was to provide results and evidence that help analyze the transmission dynamic of SARS-CoV-2 and evaluate the associations between sanitary protocols implemented, and measures to avoid SARS-CoV-2 spread in schools.

## Introduction

The Coronavirus Disease 19 (COVID-19) outbreak began in Wuhan, China in December 2019 and rapidly became an international public health emergency. As of March 2022, there had been more than 470 million cases and 6 million deaths globally [1]. The first case of COVID-19 in Spain was confirmed on January 31, 2020, and in Catalonia on February 25 [2]. Until March 2022, the Catalan region had registered more than 2 million accumulated cases and more than 26,000 deaths [3].

At the beginning of the pandemic, in March 2020, it was estimated that 107 countries and 862 million children and young people were affected by the closure of schools, one of the public health measures aiming to reduce the transmission of SARS-CoV-2 [4]. However, this number increased to 1.57 billion students worldwide over the following months [5]. Many governments chose to close schools in response to the pandemic because it has previously been shown to be an effective non-pharmacological prevention measure in the control of other virus spread like influenza [4, 6] where children have had a significantly contribution [7–9]. Nevertheless, at the beginning of the current pandemic, data on the prevalence of COVID-19 in children was scarce due to low testing of the pediatric population [10] and the fact that parameters and evidence about COVID-19 occurrence in adults could not be extrapolated to children [11]. A great deal of effort was made to resolve this question.

Since the beginning of the pandemic, the contribution of children in the virus spread has been discussed [12]. People aged from 0 to 14 had a lower risk of SARS-CoV-2 infection compared to those of 15 to 64, additionally, among the infected, older people had more severe outcomes and reported higher mortality rates [4].

Despite estimates based in household secondary attack rates may be influenced due to several factors such as contact patterns, increased exposure, and symptomatic surveillance, which depends on the sensitivity of case detection, even several serological studies estimate highest prevalence among adults under 35 years [13, 14], it is known that children are not the main source of spread of the SARS.CoV-2 virus and therefore interventions based on this public can have an impact below expected [15, 16].

The impact of school closure could also cause social, economic and health problems, with emotional costs for children and young people considering the interruptions to other areas of activity in schools such as, nutrition, mental health, safety, and social assistance services [6, 13, 14, 17–19].

In Catalonia (7,739,758 inhabitants) the closure of the 5,492 schools on 13 March 2020 affected 1,582,478 students and 116,999 teaching staff. The schools were reopened on 14 September 2020, immediately after the school vacations, between June and August, remaining closed, therefore, for six months [20].

The current schools’ guidelines were developed by the Government of Catalonia based on SARS-CoV-2 indicators. They include early detection and isolation as well implementation of public health measures as natural ventilation of classrooms, stable coexistence groups (SCG or bubble groups) and targeted screening [21, 22], also monitoring COVID-19 risk factors, determinants, transmission dynamics, preventive measures compliance and outbreaks in the school to provide evidence to improve the safety of schools preventing further closures and their impact [4, 18, 23].

The COVID-19 Sentinel Schools Network of Catalonia (CSSNC) is a part of the COVID-19 monitoring and evaluation plan from the Health Department of Catalonia. The main objective is to monitor and evaluate the epidemiological situation of COVID-19 and its determinants in the educational setting, to gather evidence for the health policies aimed at the prevention and control of SARS-CoV-2 infection in schools and as a platform for other applied research projects. Currently, in 2022, the CSSNC includes 23 schools with 4,221 children and 1,140 staff from all over Catalonia, the study protocol has been previously published [24].

The aims of the CSSNC project (www.escolessentinella.org) include: the monitoring of biological markers; knowledge, attitudes and behaviors towards SARS-CoV-2 preventive measures; the identification of both facilitators and barriers to their implementation; and the monitoring of environmental indicators such as CO2, all together using a participatory research approach [24].

In this paper, to answer the question about the occurrence of COVID-19 among school-aged population, the main objectives are to estimate the prevalence and incidence of SARS-CoV-2 exposure and infections and to identify potential associated factors associated to them, among students and staff of the CSSNC during the academic year 2020–2021. Moreover, as a secondary objective, feasibility of a by-monthly testing strategy is also assessed.

## Materials and methods

### Study design and population

In this study, were included 2,007 students and 520 school staff (teaching and non-teaching staff, such as extracurricular education instructors and administrative personal) who previously signed the informed consent, from seven schools all over Catalonia. Although they are an opportunistic sample, epidemiological, and sociodemographic characteristics of the area, as well as type of school (public, private or chartered) were considered to assure heterogeneity. During the study period, we used two methodological approaches: a cross-sectional study to estimate SARS-CoV-2 prevalence, and a longitudinal study to calculate the COVID-19 incidence and evaluate the feasibility of the twice-monthly testing strategy.

The cross-sectional component included students aged 3–19 from preschool (3–5 year-old), elementary school (7–10 year-old), middle school (12–15 year-old), high school (16–17 year-old) and vocational training (17–19 year-old) and school staff.

For the longitudinal component, were included in a cohort of 1,424 participants, 983 students over 12 years-old and 441 school staff.

### Data collection

**Fig 1:**
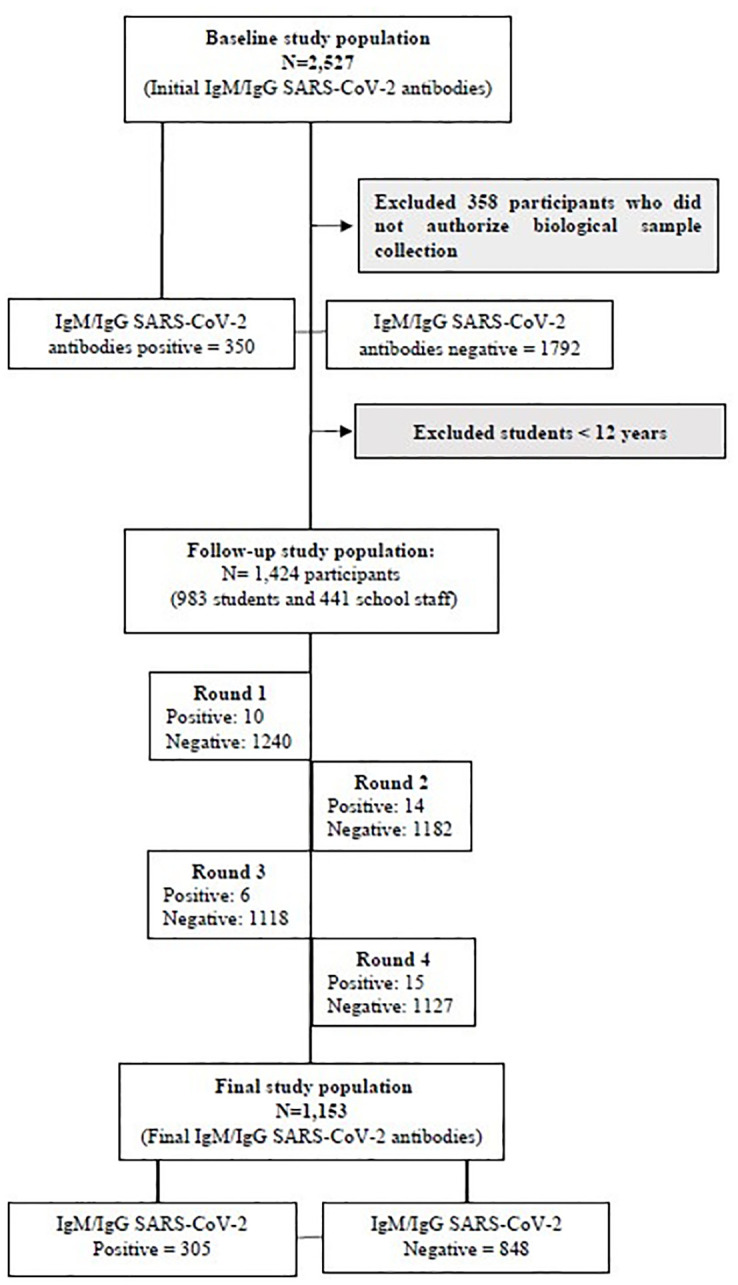
Flowchart of population at cross-sectional and longitudinal prospective components of the study.

The cross-sectional component was performed between February 22 and March 22, 2021, and the longitudinal component, were proceeded through 4 data collection rounds, between 6–19 April 2021, 20 April 2021 and 03 May 2021, 04 May 2021 and 18 May 2021 being the last round between 19 May 2021 and 02 June 2021 (Fig 1).

We collected nominal data from an online questionnaire but, when necessary, a paper form was used. Questions about demographic and economic characteristics, health status, knowledge, perceptions and behaviors related to COVID-19, control measures, pandemic impacts, previous SARS-CoV-2 infection, symptoms and contacts pattern as health indicators were included according to COSMO questionnaire [25]. In each longitudinal round, participants filled in an additional online epidemiological survey with information related to SARS-CoV-2 infection, suspected symptoms, exposures, and vaccine status during the previous 15 days.

Three different questionnaire models were designed one for school staff (questionnaire A); one for students under 16 years, which were answered by parents/guardian (questionnaire B) and one for students over 16 years (questionnaire C).

Secondary data about vaccine coverage and socioeconomic level was provided by the Agència de Qualitat I Avaluació Sanitàries de Catalunya (AquAS) through the Primary Care Services Information System (SISAP) and Data Analytics Program for Health Research and Innovation (PADRIS), which collect programmatic data from different sources. The variable socioeconomic level was based on the sanitary regions and was used to categorize the place of residences in tertiles (high, medium and low).

Biological samples were collected from all participants. A finger prick blood was collected to perform a rapid serological anti-SARS-CoV-2 IgM/IgG test to estimate the initial and final seroprevalence in February and June 2021 respectively. Saliva and nasal swab were collected twice a month to investigate the presence of SARS-CoV-2 RNA and SARS-CoV-2 antigens.

All results were uploaded to the electronic health record, and, become available to the participants normally within 48 hours of the sample collection.

### Independent variables

Factors that could have impact in outcome were referred to as independent variables, that were tested to investigate the association with the SARS-CoV-2 infection. They were categorized in sociodemographic, health status, contact patterns, knowledge and perceptions and, preventive measures. Each variable was coded according to the type of the question asked in the questionnaire (Table 1).

**Table 1:** Independent variables included in the study, CSSNC Catalonia, Spain 2021.

**Sociodemographic**	
Sex	Male / Female
Age	Years [IQR]
Parents’ occupation	Dichotomized: Employed / unemployed, retired and lay-of
Parents’ or school staff level of completed studies	Higher studies or university / Secondary school / Primary school or None
House size (in meters)	Dichotomized: >70m2 / <70m2
Economic situation	Changed to worse / improved
Socioeconomical status	Low / middle / high
Parents or Staff changed employment status during the pandemic	yes / no
**Contact pattern**	
Contact with suspected or confirmed COVID-19 cases (Unspecific)	yes / no
Place of contact with suspected or confirmed COVID-19 cases	Home / school / other
Living with a healthcare professional	yes / no
Main mode of transport	On foot / bicycle / own motorcycle or car / public transport / school bus
Avoiding contact in crowded spaces	Likert 5-point scale dichotomized: No (never, almost never or sometimes) Yes (most of the time or always)
**Health Status**	
Self-reported health status	Likert 7-point scale dichotomized: Low (1–4) / High (5–7)
Underlying medical conditions	yes / no
Specific underlying medical conditions	Hypertension / Asthma / Obesity / Diabetes mellitus / Chronic heart disease
**Knowledge and perceptions**	
Perceived knowledge	Likert 5-point scale dichotomized: No (never, almost never or sometimes) Yes (most of the time or always)
Perception of risk	Likert 7-point scale dichotomized: Low (1–4) / High (5–7)
**Compliance of preventive measures in the last 7 days**	
Washing hands	yes / no
Avoiding close contact with someone who is infected or at risk	yes / no
Avoiding crowded spaces or crowds	yes / no
Avoiding closed or indoor spaces	yes / no
Ventilating closed spaces whenever possible	yes / no
Wearing a mask	yes / no
Self-isolation	yes / no
Frequently disinfecting used objects	yes / no
Avoiding public transportation	yes / no
Use of hand sanitizing gels	yes / no
Avoiding touching the face, eyes, mouth with unwashed hands	yes / no
Staying at home if I have a cold or other illness	yes / no
Avoiding trips abroad	yes / no
Covering the mouth with the elbow when coughing or sneezing	yes / no

### Laboratory assays

A RT-PCR assay (Allplex SARS-CoV-2/FluA/FluB/RSV, Werfen, Korea) and a molecular assay based on the transcription mediated amplification assay (TMA) (Procleix SARS-CoV-2, Grifols, Barcelona, Catalonia, Spain) was conducted to detect SARS-CoV-2 RNA. The nasal swab samples for detection of SARS-CoV-2 antigen were processed using the Panbio COVID-19 Ag Rapid Test (Abbot, Chicago, IL, USA), following the manufacturer’s instructions, with a sensitivity of 93.3% (95% CI: 83.8–98.2%) and specificity of 99.4% (95% CI: 97.0–100%). For the anti-SARS-CoV-2 IgM/IgG test we used a rapid SARS-CoV-2 serological test (COVID-19 IgG/IgM Rapid Test Kit, Lambra, Madrid, Spain), following the manufacturer’s instructions, with sensitivities of 97.2% (IgG) and 87.9% (IgM), and specificities of 100% for both immunoglobulins, but following recommendations to the Public Health Protocol for COVID-19 [22], because of the low specificity of IgM antibodies and several reported cross-reactions with other non-specifics proteins, only IgG antibodies were used to assess the prevalence ofSARS-CoV-2.

The samples that SARS-CoV-2 were detected were stored in the sample collection C.0001145 located at the Vall d’Hebron Hospital Universitari (Barcelona, Spain) in the sample collection registered at the Instituto de Salud Carlos III register, Madrid, Spain.Saliva samples with positive SARS-CoV-2 results were frozen and stored at the IGTP-HUGTiP Biobank, Badalona, Catalonia, Spain, and maintained for two years.

### Outcomes and case definitions

Our first outcome was previous exposure to SARS-CoV-2 virus. The case definition for positive, was any individual with a positive SARS-CoV-2 IgG antibodies detected by rapid test.

The second outcome was active SARS-CoV-2 infections. The case definition for positive was any individual, symptomatic or asymptomatic with a positive RT-PCR or RAT detected by the project team or detected and confirmed by RT-PCR or RAT performed by primary health care, during the follow-up period. We decided to include these self-reported documented infections because students and school staff with positive results started the isolation protocol and were no longer tested at school.

### Data analysis

We calculated crude and adjusted prevalence for students of 2–20 years of age on the census in Catalan schools, adjusting for age and sex. For school staff, we only adjusted for sex and then by sensitivity and specificity of the tests. The differences between initial and final seroprevalence were only calculated for students from first grade of middle school or older and staff, using the McNemar test.

Descriptive analysis was performed, and the data were provided globally and stratified by educational stage when possible and presented considering sociodemographic and socioeconomic indicators; contact pattern; knowledge, behavior and perceptions of COVID-19 and health status. Frequency, measures of central tendency (mean and median) and dispersion (standard deviation and IQR) were calculated.

Univariate analysis was performed to investigate the association between independent variables and outcome, variables with p-value <0.050 were considered statistically significant. The prevalence ratio with 95% confidence interval (CI) were calculated using an adjusted Poisson model with robust error and adjustment for age, sex and school. The combined qualitative variables were compared using the McNemar test.

For the multivariate analysis, we proceeded an initial correlation graph (polychoric correlation) was constructed for each category of variables and of the pairs that had a correlation coefficient greater than 0.8 (in absolute value), only one of the variables was used for the analysis. To fit a multivariate model, we performed the analysis with stepforward and stepwise regression, ie. starting with the model with all variables, then removing them one by one and subsequently starting with an empty model adding the variables one by one. We used the same type of regression as for univariate models (GLM Poisson with robust errors). We selected variables by significance, R2 and AIC, and both models (stepforward and stepwise regression) gave us the same model with R2 0.184547 and AIC 1261.318, compatible with the set of behavioral variables included in the model. We checked the Goodness of fit for the multivariate model and observed that no overdispersion was found in the Poisson model.

To calculate the incidence rate for the at-risk population, we used the number of participants with a positive result in RT-PCR or RAT divided by person time at risk. For at risk of infection we excluded individuals with RT-PCR or RAT positive result in the previous 60 days before started the cohort. The denominator was defined as the sum of the time at risk of the 1,366 participants sampled during the cohort (950 students and 416 employees). Time at risk was defined for each participant, as the difference of time between the moment that they entered the study and the endpoint when they tested positive or, if they did not obtain any positive result, the last round that they were tested. The result was presented *per 100 person-month*.

In the longitudinal component, a univariate analysis was performed included the same variables that had been tested in the cross-sectional component, adding the information collected during the follow-up endpoints. These data were assessed using independent log binomial mixed models to calculate the Relative Risk (RR) with participant’s identifier as random effect and adjusting for age, sex and school. Due the low number of positives, a multivariate model for the longitudinal component was not proceed.

For the univariate analysis, a GLMM log binomial model with ID as random intercept was used to estimate the PR and RR of each variable of interest, by means of an adjusted measure and avoiding the confusion of the variables age, sex and school.

Two composite indicators were created to measure the participant’s knowledge of COVID-19: “*perceived knowledge*” and “*factual knowledge*” and another indicator to measure the “*risk perception”*. The “*perceived knowledge*” and “*risk perception*” were measured using a Likert 7-point scale, 1–4 scores were considered as *low* level of knowledge or *low* level of risk perception and 5–7 as *high* level. And “*factual knowledge*” was measured by a binary score composed of three aspects: people at risk, symptoms and means of transmission. The answers were counted, and we classified as *high* level of *factual knowledge* when more than 50% of answers were correct and 50% or less as *low*.

All analyses were carried out with R (version 4.1.0). Confidence intervals for incidence were obtained using the ‘epi.conf’ function from ‘epiR’ package The number of samples that should be tested to find a positive was calculated using Ene 3.0.

### Ethics statement

The Foundation University Institute for Research in Primary Health Care Jordi Gol i Gurina (IDIAPJGol) approved the study on 17 December 2020 (code 20/192-PCV). Informed consent was obtained from school staff, parents for those children under 16 and alumni aged 16 or older. Participants were free to decline/withdraw consent at any time without providing a reason and without being subject to any resulting detriment.

## Results

For participants students, except for the preschool, most participants were female (55%) overall. Regarding socioeconomic variables, 821 (41.7%) students’ fathers and 1,043 mothers (52.3%) have high levels of study or university qualifications, 1,705 (86.7%) of the students’ fathers and 1613 (81.0%) of the students’ mothers were employed in the study period (Table 2).

**Table 2:** General characteristics of the students participating in the sentinel school project, Catalonia, Spain. February-March 2021.

Variables	Preschool		Elementary school		Middle school		High school		Vocational Training	
	N = 223		N = 752		N = 599		N = 316		N = 117	
Sociodemographic and socioeconomic indicators	n	%	n	%	n	%	n	%	n	%
Sex										
Female	108	48.4%	387	51.5%	306	51.1%	211	66.8%	92	78.6%
Male	115	51.6%	365	48.5%	293	48.9%	105	33.2%	25	21.4%
Age (years)	4.00 [3.00;5.00]		9.00 [7.00;10.0]		13.0 [12.0;15.0]		17.0 [16.0;17.0]		18.0 [17.0;19.0]	
Level of completed studies (father)										
Without formal education or incomplete primary Education	10	4.6%	36	5,00%	22	3.7%	4	1.3%	14	12,00%
Primary school certificate	38	17.4%	140	19.2%	135	22.9%	44	13.9%	40	34.2%
Secondary school certificate	63	28.8%	208	28.6%	159	26.9%	93	29.4%	33	28.2%
Higher studies or University	105	47.9%	329	45.2%	224	38,00%	154	48.7%	9	7.7%
Don’t know	0	0.0%	2	0.3%	33	5.6%	18	5.7%	16	13.7%
Not applicable	3	1.4%	13	1.8%	17	2.9%	3	1,00%	5	4.3%
Level of completed studies (mother)										
Without formal education or incomplete primary Education	11	5.0%	43	5.8%	27	4.6%	4	1.3%	12	10.3%
Primary school certificate	22	10.0%	76	10.2%	91	15.3%	28	8.9%	40	34.2%
Secondary school certificate	42	19.1%	193	25.8%	168	28.3%	88	27.8%	34	29.1%
Higher studies or University	144	65.5%	427	57.2%	275	46.4%	182	57.6%	15	12.8%
Don’t know	0	0.0%	4	0.5%	26	4.4%	12	3.8%	14	12,00%
Not applicable	1	0.5%	4	0.5%	6	1,00%	2	0.6%	2	1.7%
Occupation (father)										
Not applicable	8	3.7%	27	3.7%	39	6.6%	7	2.2%	12	10.3%
Others	2	0.9%	17	2.3%	8	1.4%	1	0.3%	3	2.6%
Retired	0	0.0%	5	0.7%	10	1.7%	7	2.2%	8	6.8%
Household keeper	2	0.9%	1	0.1%	0	0.0%	0	0.0%	0	0.0%
Unemployed	5	2.3%	17	2.3%	38	6.5%	9	2.9%	4	3.4%
Sick leave	3	1.4%	7	1.0%	9	1.5%	6	1.9%	6	5.1%
Employed	198	90.8%	652	89.8%	485	82.3%	286	90.5%	84	71.8%
Occupation (mother)										
Not applicable	5	2.3%	33	4.4%	23	3.9%	7	2.2%	3	2.6%
Others	4	1.8%	12	1.6%	8	1.4%	1	0.3%	3	2.6%
Retired	1	0.5%	0	0.0%	4	0.7%	1	0.3%	2	1.7%
Household keeper	19	8.7%	42	5.6%	56	9.4%	15	4.8%	14	12.0%
Unemployed	11	5.1%	29	3.9%	37	6.2%	6	1.9%	7	6.0%
Sick leave	3	1.4%	12	1.6%	7	1.2%	5	1.6%	8	6.8%
Employed	175	80.3%	618	82.8%	459	77.3%	281	88.9%	80	68.4%
House size (m2)										
<50 m2	1	0.5%	8	1.1%	12	2.0%	4	1.3%	0	0.0%
51–70 m2	22	10.0%	61	8.1%	57	9.6%	13	4.1%	10	8.6%
71–90 m2	85	38.5%	235	31.4%	131	22.0%	70	22.2%	28	23.9%
91–110 m2	48	21.7%	190	25.4%	132	22.2%	67	21.2%	17	14.5%
111–130 m2	35	15.8%	111	14.8%	93	15.6%	54	17.1%	15	12.8%
>130 m2	28	12.7%	142	19.0%	135	22.7%	76	24.1%	19	16.2%
Don’t know	2	0.9%	4	0.5%	36	6.1%	49	15.5%	34	29.1%
Underlying medical conditions										
No	205	93.2%	698	93.4%	538	90.1%	289	91.5%	97	82.9%
Yes	15	6.8%	49	6.6%	59	9.9%	27	8.5%	20	17.1%
Specific underlying medical conditions (n = 274)										
Hypertension	0	0.0%	0	0.0%	0	0.0%	1	3.7%	0	0.0%
Asthma	5	33.3%	12	25.5%	31	52.5%	8	29.6%	9	45.0%
Obesity	0	0.0%	0	0.0%	1	1.7%	0	0.0%	0	0.0%
Diabetes mellitus	0	0.0%	1	2.1%	3	5.1%	1	3.7%	1	5.0%
Chronic heart disease	2	13.3%	3	6.38%	1	1.7%	1	3.7%	0	0.0%

For school staff, mean [IQR] age was 43 [IQR 33–51], 410 (78.8%) were female, 418 (80.5%) had a high level of studies or university qualifications and 292 (56.2%) used their own car as the main means of transport. About 20% (104) had a comorbidity, the most common was asthma 23 (22.1%) and hypertension 21 (20.2%) (Table 3).

**Table 3:** General characteristics of the school staff participating in the sentinel school project, Catalonia, Spain. February-March 2021.

Variables	School staff	
	N = 520	
Sociodemographic and socioeconomic indicators	n	%
Sex		
Female	410	78.8%
Male	110	21.2%
Age (years)	43.0 [33.0;51.0]	
Level of completed studies (school staff)		
Without formal education or incomplete primary Education	4	0.8%
Primary school certificate	28	5.4%
Secondary school certificate	69	13.3%
Higher studies or University	418	80.5%
House size (m2)		
<50 m2	15	2.9%
51–70 m2	71	13.7%
71–90 m2	143	27.5%
91–110 m2	112	21.5%
111–130 m2	72	13.8%
>130 m2	91	17.5%
Don’t know	16	3.1%
**Health status**		
Underlying medical conditions		
No	416	80.0%
Yes	104	20.0%
Specific underlying medical conditions (n = 274)		
Hypertension	21	20.2%
Asthma	23	22.1%
Obesity	8	7.7%
Diabetes mellitus	4	3.9%
Chronic heart disease	2	1.9%

Regarding epidemiological data for SARS-CoV-2 exposure risk, for students, the most common place of contact with suspected or confirmed case was at the school, being 65 (65.0%) in preschool group for school staff, 122 (63.2%) reported having contact with a suspected or confirmed case at the school (Table 4).

**Table 4:** Contact pattern of students and school staff participating in the sentinel school project, Catalonia, Spain. February-March 2021.

Variables	Preschool		Elementary school		Middle school		High school		Vocational Training		School staff	
	N = 223		N = 752		N = 599		N = 316		N = 117		N = 520	
Lives with healthcare professional												
No	189	85.1%	656	87.6%	538	90.6%	274	86.7%	104	88.9%	442	92.9%
Yes	33	14.9%	93	12.4%	56	9.4%	42	13.3%	13	11.1%	34	7.1%
Main mode of transport												
On foot	152	68.2%	495	65.8%	378	63.3%	136	43.0%	22	18.8%	152	29.2%
Scooter/bicycle	30	13.5%	30	4.0%	29	4.9%	33	10.4%	1	0.9%	36	6.9%
Motorcycle/own car	81	36.3%	284	37.8%	173	29.0%	119	37.7%	71	60.7%	292	56.2%
Public transport	21	9.4%	89	11.8%	106	17.8%	103	32.6%	39	33.3%	97	18.7%
School bus	1	0.5%	5	0.7%	3	0.5%	1	0.3%	1	0.9%	0	0.0%
Contact with suspected or confirmed COVID-19 cases												
Don’t know	72	32.6%	320	43.0%	155	26.1%	54	17.1%	22	18.8%	205	39.5%
No	48	21.7%	174	23.4%	168	28.2%	100	31.6%	24	20.5%	121	23.3%
Yes, with both confirmed and suspected cases	4	1.8%	22	3.0%	26	4.4%	23	7.3%	12	10.3%	40	7.7%
Yes, with suspected cases	5	2.3%	13	1.8%	16	2.7%	13	4.1%	4	3.4%	14	2.7%
Yes, with confirmed cases	92	41.6%	215	28.9%	230	38.7%	126	39.9%	55	47.0%	139	26.8%
Place of contact												
At home	42	42.0%	122	49.6%	115	42.3%	72	44.4%	23	32.4%	58	30.1%
At school	65	65.0%	137	55.7%	165	60.7%	82	50.6%	41	57.7%	122	63.2%
Leisure activities	2	2.0%	25	10.2%	53	19.5%	54	33.3%	17	23.9%	26	13.5%
Don’t know	0	0.0%	3	1.2%	0	0.0%	8	4.9%	4	5.6%	0	0.0%
Others	5	5.0%	9	3.7%	17	6.3%	11	6.8%	16	22.5%	23	11.9%

### Seroprevalence, univariate and multivariate analysis in the cross-sectional component

The baseline seroprevalence of SARS-CoV-2 IgG for students and school staff was, respectively, 14.8% (95% CI: 13.1–16.5) and 22% (95% CI: 18.3–25.8); adjusted for sensitivity and specificity it was 15.2% (95% CI: 13.5–17) and 22.6% (95% CI: 19–26.7) and the weighted seroprevalence was 14.5% (95% CI: 12–17.1) and 22.0% (95% CI: 21.2–22.8). The seroprevalence of SARS-CoV-2 IgG at the end of the longitudinal component for students over 12 years-old and staff was 18.4% (95% CI: 15.6–21.1) and 42.6% (95% CI: 37.7–47.5), adjusted for sensitivity and specificity it was 18.9% (95% CI: 16.2–21.9) and 43.8% (95% CI: 38.8–49.0) and the weighted seroprevalence was 19.7% (95% CI: 13.3–26.1) and 42.5% (95% CI: 0–92.4). The prevalence of active SARS-CoV-2 infection confirmed by RT-PCR or RAT at baseline was 0.7% (95% CI: 0.3–1.0) in students and 1.1% (95% CI: 0.1–2.0) in school staff. Weighted prevalence was 1% (95% CI: 0–2.1) and 1.1% (95% CI: 1–1.1). Self-reported documented infection of SARS-CoV-2 between February 2020 and March 2021 was 8.5% (95% CI: 7.3–9.8) for students and 7.1% (95% CI: 4.9–9.3) for staff. For students, when weighted by sex and age it was 8.2% (95% CI: 4.3–12.2) and for staff when weighted by sex it was 7.1% (95% CI: 5.9–8.4) (Table 5).

**Table 5:** Summary of crude and adjusted seroprevalence and active infections during the first trimester of 2021 in sentinel school project, Catalonia, Spain.

Indicator	Students n = 2007	Staff n = 520
	% (95%CI)	% (95%CI)
**Seroprevalence**		
Initial crude seroprevalence of SARS-CoV-2 IgG	14.8 (13.1–16.5)	22 (18.3–25.8)
Adjusted initial seroprevalence for sensitivity and specificity ^1^	15.2 (13.5–17)	22.6 (19–26.7)
Weighted initial seroprevalence of SARS-CoV-2 IgG ^2^	14.5 (12–17.1)	22.0 (21.2–22.8)
Final crude seroprevalence of SARS-CoV-2 IgG (n = 1153)	18.4 (15.6–21.1)	42.6 (37.7–47.5)
Adjusted final seroprevalence for sensitivity and specificity ^1^ (n = 1153)	18.9 (16.2–21.9)	43.8 (38.8–49.0)
Weighted final seroprevalence of SARS-CoV-2 IgG ^2^ (n = 1153)	19.7 (13.3–26.1)	42.5 (0–92.4)
**Active infection**		
Prevalence of active SARS-CoV-2 infection at baseline ^3^	0.7 (0.3–1.0)	1.1 (0.1–2.0)
Weighted prevalence of active SARS-CoV-2 infection at baseline ^2^^,^ ^3^	1 (0–2.1)	1.1 (1–1.1)

^1^.COVID-19 IgG/IgM Rapid Test Kit, Lambra with sensitivities of 97,2% (IgG) and 87,9% (IgM), and specificities of 100%

^2^. Weighted according to 2020 student censused in Catalonia age 2–20 years by sex and age and school staff by sex. Estimated prevalence of the biological samples collected by the project team or the applied questionnaire

^3^. Biological samples collected during baseline, TMA with nasal swab performed by project team between Feb and Mar 2021

Abbreviations: IgG: immunoglobulin G; 95% CI: 95% Confidence interval

**Fig 2:**
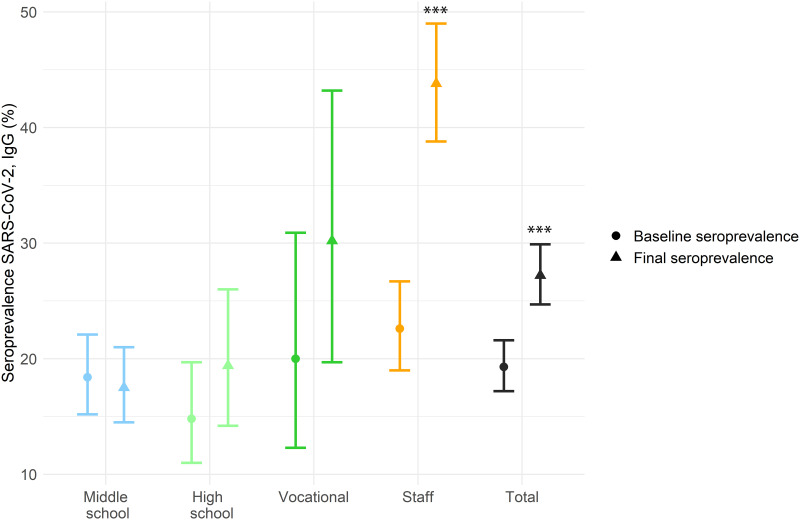
Adjusted seroprevalence of antibodies IgG anti-SARS-CoV-2 in longitudinal population by educational stages and school staff at the baseline (February-March 2021) and the last follow-up (May-June 2021). *** p <0.001 using McNemar test.

Among those participants who had two serological tests at baseline and at the end of the longitudinal component (round four), there was a significant increase in prevalence (p<0.001). The main differences were in the staff group (p-value <0,001), although there was also a no significant increase among students in vocational studies (Fig 2).

The variables included in the univariate analysis, for students and staff were presented into sociodemographic, health and behaviors and contact patterns categories. Indiscriminate *changes in the employment situation* (PR 1.43, CI 1.07–1.91) and *improved the economic situation* (PR 2.66 CI 1.18–6.00) regarding parents and school staff were positively associated with having been infected. The variable higher *perceived knowledge* was positively associated with the infection (PR 1.68 CI 1.05–2.68). The public health measure, *avoiding crowded spaces* was negatively associated with the infection (PR 0.65 CI 0.45–0.93) and a higher *risk perception* was positively associated (PR 1.49 CI 1.14–1.93) (Table 6).

**Table 6:** Summary and univariate analysis between SARS-CoV-2 infection and sociodemographic, health and behavioral indicators of students and staff from sentinel schools. Catalonia, February to March 2021.

Variables	Students				Staff				Total				Univariate analysis	
	Positive N = 180		Negative N = 1827		Positive N = 41		Negative N = 479		Positive N = 221		Negative N = 2306		Prevalence ratio	p-value
	n	%	n	%	n	%	n	%	n	%	n	%	(PR)	
**Sociodemographic**														
Sex													-	-
Female	101	56.1	1003	54.9	32	78.0	378	78.9	133	60.2	1381	59.9	-	-
Male	79	43.9	824	45.1	9	22.0	101	21.1	88	39.8	925	40.1	-	-
Age (years)	15.0 [11.0;16.0]		12.0 [8.00;15.0]		45.0 [32.0;50.0]		43.0 [33.5;51.0]		15.0 [12.0;18.0]		13.0 [9.00;17.0]		-	-
Changed employment status during the pandemic														
No	128	73.1	1418	79.7	34	82.9	392	82.7	162	75.0	1810	80.3	ref	
Yes	47	26.9	362	20.3	7	17.1	82	17.3	54	25.0	444	19.7	1.43 (1.07–1.91)	0.015
Economic situation														
Changed to worse	40	88.9	407	96.4	3	60.0	69	82.1	43	86.0	476	94.1	ref	
Improved	5	11.1	15	3.6	2	40.0	15	17.9	7	14.0	30	5.9	2.66 (1.18–6.00)	0.019
Socioeconomical status														
Low	77	42.8	837	46.5	23	56.1	239	51.1	100	45.2	1076	47.4	ref	
Middle	43	23.9	542	30.1	13	31.7	132	28.2	56	25.3	674	29.7	0.74 (0.52–1.06)	0.098
High	60	33.3	422	23.4	5	12.2	97	20.7	65	29.4	519	22.9	1.12 (0.76–1.65)	0.582
**Knowledge and perceptions**														
Perceived knowledge **														
Low	13	24.1	150	37.8	7	17.1	135	28.2	20	21.1	285	32.5	ref	
High	41	75.9	247	62.2	34	82.9	344	71.8	75	78.9	591	67.5	1.68 (1.05–2.68)	0.030
Risk perception**														
Low	120	66.7	1435	79.2	17	41.5	228	47.6	137	62.0	1663	72.6	ref	
High	60	33.3	377	20.8	24	58.5	251	52.4	84	38.0	628	27.4	1.49 (1.14–1.93)	0.003

* Likert scale. No: never, almost never or sometimes; Yes: most of the time or always

** Likert scale. Low: 1–4; High: 5–7

*** GLM adjusted Poisson model with robust error and adjustment for age, sex and school

In the case of contact patterns, *having unspecific contact with suspected or confirmed COVID-19 case*, (PR 2.76; CI 1.94–3.93) or contact at home (PR 2.17; CI 1.62–2.91) were positively associated with the infection, in contrast, contact at school, had a strongly negative association (PR 0.60 CI 0.45–0.80). *Living with a health professional* was not associated with infection (p-value = 0.262). (Table 7).

**Table 7:** Univariate analysis between SARS-CoV-2 infection and contact patterns of students and staff from sentinel schools. Catalonia, Spain, February-March 2021.

Variables	Students				Staff				Total				Univariate analysis	
	Positive N = 180		Negative N = 1827		Positive N = 41		Negative N = 479		Positive N = 221		Negative N = 2306		Prevalence ratio	p-value
	n	%	n	%	n	%	n	%	n	%	n	%		
Lives with healthcare professional														
No	163	91.1	1598	87.9	35	94.6	407	92.7	198	91.7	2005	88.8	ref	
Yes	16	8.9	221	12.1	2	5.4	32	7.3	18	8.3	253	11.2	0.77 (0.49–1.22)	0.262
Unspecific contact with suspected cases of COVID-19														
Don’t know	24	13.4	599	33.0	13	31.7	192	40.2	37	16.8	791	34.5	ref	
No	21	11.7	493	27.2	4	9.8	117	24.5	25	11.4	610	26.6	0.79 (0.49–1.29)	0.349
Yes	134	74.9	722	39.8	24	58.5	169	35.4	158	71.8	891	38.9	2.76 (1.94–3.93)	<0.001
Contact with suspected cases of COVID-19 at home														
No	52	38.8	425	59.3	12	50.0	123	72.8	64	40.5	548	61.9	ref	
Yes	82	61.2	292	40.7	12	50.0	46	27.2	94	59.5	338	38.1	2.17 (1.62–2.91)	<0.001
Contact with suspected cases of COVID-19 at school														
No	66	49.3	295	41.1	15	62.5	56	33.1	81	51.3	351	39.6	ref	
Yes	68	50.7	422	58.9	9	37.5	113	66.9	77	48.7	535	60.4	0.60 (0.45–0.80)	<0.001
Avoiding contact in crowded spaces*														
No	26	14.4	170	9.5	4	10.0	23	4.9	30	13.6	193	8.5		
Yes	154	85.6	1625	90.5	36	90.0	452	95.2	190	86.4	2077	91.5	0.65 (0.45–0.93)	0.019
Self-reported health status**														
Low	8	4.4	27	1.5	2	4.9	22	4.6	10	4.5	49	2.1	ref	
High	172	95.6	1794	98.5	39	95.1	457	95.4	211	95.5	2251	97.9	0.55 (0.30–1.01)	0.053

* Likert scale. No: never, almost never or sometimes; Yes: most of the time or always

** Likert scale. Low: 1–4; High: 5–7

*** GLM adjusted Poisson model with robust error and adjustment for age, sex and school

In the multivariate analysis, the same significance and trend among socioeconomic, health measures and contact patterns variables were observed (Table 8).

**Table 8:** Multivariate analysis between SARS-CoV-2 infection, sociodemographic and health indicators, by students and staff from sentinel schools. Catalonia, February to March 2021.

Variables	Estimate	CI_low	CI_upp	p-value
Contact pattern	2.205	1.876	2.591	<0.001
Perceived gravity if infected with coronavirus	1.328	1.011	1.744	0.041
Be employed	1.348	1.009	1.801	0.043
Compliance of sanitary measures	0.547	0.372	0.805	0.002
Self-reported health status	0.432	0.221	0.845	0.014
Use of transport public or school bus	0.522	0.327	0.836	0.007
Parents educational level (higher)	0.726	0.554	0.953	0.021

### Incidence and univariate analysis at the longitudinal component

During the longitudinal component of the study, 45 new infections occurred (34 students and 11 staff), 11 of them identified by RT-PCR performed by the project team and 34 self-reported in by the participants. It is interesting to note that out of 11 RT-PCR positives identified in the study, only 1 (9%) was also detected by RAT.

The overall incidence was 2.73 (95% CI 1.991, 3.653) per 100 person-month, that is 2.887 (95% CI 1.999, 4.034) and 2.337 (95% CI 1.167, 4.182) per 100 person-month for students and staff, respectively.

The variables included in the univariate analysis were also categorized into sociodemographic and socioeconomic indicators, health status, and preventive compliance. There was a protective behavior associated with socioeconomic status, when comparing the highest level (high) in reference to the first tercile (low) (RR 0.25, 95% CI 0.06–0.96) for the COVID-19 infection (Table 9).

**Table 9:** Longitudinal component results by follow-up endpoint and relative risk adjusted by sex and age, of risk factors for SARS-CoV-2 infection in participants of the longitudinal component. Sentinel schools project, Catalonia, Spain, April to June 2021.

Variables	Round 1				Round 2				Round 3				Round 4				Univariate analysis	
	Positive N = 10		Negative N = 1250		Positive N = 14		Negative N = 1182		Positive N = 6		Negative N = 1118		Positive N = 15		Negative N = 1127		RR (95% CI)	p-value
	n	%	n	%	n	%	n	%	n	%	n	%	n	%	n	%		
**Socioeconomic**																		
Sex																		
Female	4	40.0	815	65.2	5	35.7	761	64.4	5	83.3	709	63.4	10	66.7	722	64.1	-	-
Male	6	60.0	435	34.8	9	64.3	421	35.6	1	16.7	409	36.6	5	33.3	405	35.9	-	-
Age—years [IQR]	15.5 [14.2;16.8]		16.0 [14.0;31.0]		15.0 [13.0;16.0]		16.0 [14.0;32.0]		14.5 [12.5;34.5]		16.0 [13.0;32.0]		16.0 [14.0;34.5]		16.0 [14.0;34.0]		-	-
Socioeconomic level																		
Low	5	50.0	657	53.2	10	71.4	629	53.9	2	33.3	553	50.1	8	53.3	587	52.9	ref	
Middle	4	40.0	351	28.4	3	21.4	319	27.4	4	66.7	336	30.4	6	40.0	324	29.2	0.91 (0.43–1.93)	0.814
High	1	10.0	226	18.3	1	7.14	218	18.7	0	0.0	215	19.5	1	6.7	199	17.9	0.25 (0.06–0.96)	0.044
**Contact pattern** *																		
Had contact																		
Don’t know	3	33.3	607	59.4	4	36.4	452	60.6	2	33.3	247	60.4	8	66.7	341	69.0	ref	
No	4	44.4	347	34.0	2	18.2	247	33.1	0	0.0	132	32.3	2	16.7	134	27.1	0.74 (0.32–1.75)	0.497
Yes	2	22.2	68	6.7	5	45.5	47	6.3	4	66.7	30	7.33	2	16.7	19	3.9	6.44 (3.15–13.19)	<0.001
Contact at home																		
No	8	88.9	1001	97.9	8	72.7	742	99.5	4	66.7	400	97.8	11	91.7	492	99.6	ref	
Yes	1	11.1	21	2.1	3	27.3	4	0.5	2	33.3	9	2.2	1	8.3	2	0.40	12.42 (5.81–26.52)	<0.001
Contact at school																		
No	8	88.9	985	96.4	10	90.9	710	95.2	4	66.7	396	96.8	11	91.7	479	97.0	ref	
Yes	1	11.1	37	3.6	1	9.1	36	4.8	2	33.3	13	3.2	1	8.3	15	3.0	3.73 (1.49–9.38)	0.005
Unspecific contact																		
No	8	88.9	990	96.9	9	81.8	729	97.7	4	66.7	400	97.8	12	100	490	99.2	ref	
Yes	1	11.1	32	3.1	2	18.2	17	2.3	2	33.3	9	2.2	0	0.0	4	0.8	5.28 (2.1–13.27)	<0.001
**Health status**																		
Chronic diseases																		
No	6	60.0	1083	86.8	13	92.9	1023	86.7	3	50.0	965	86.5	12	80.0	979	87.0	ref	
Yes	4	40.0	165	13.2	1	7.1	157	13.3	3	50.0	151	13.5	3	20.0	146	13.0	2.18 (1.1–4.33)	0.026
**Preventive measures** **																		
Wearing a mask																		
No	0	0.0	8	0.8	1	10.0	5	0.7	1	16.7	1	0.3	0	0.0	1	0.2	ref	
Yes	9	100	1012	99.2	9	90.0	741	99.3	5	83.3	404	99.8	11	100	490	99.8	0.14 (0.04–0.53)	0.004
Avoid contact*																		
No	2	22.2	43	4.5	1	10.0	39	5.6	1	16.7	20	5.3	1	9.1	25	5.5	ref	
Yes	7	77.8	921	95.5	9	90.0	656	94.4	5	83.3	361	94.8	10	90.9	429	94.5	0.38 (0.15–0.97)	0.043

* With people suspect, confirmed or at risk for COVID-19

** In the last 7 days

*** Calculated using an independent log binomial mixed model

C*ontact with a suspected or confirmed case of COVID-19*, was a risk factor for infection (RR 6.44, 95% CI 3.15–13.19). When the contact occurred at home, the risk (RR 12.42, 95% CI 5.81–26.52) was higher than compared to school (RR 3.73, 95% CI 1.49–9.38) and other non-specific locations (RR 5.28, 95% CI 2.1–13.27).

We tested several sanitary measures that had been carried out in the last seven days before the survey and only *avoiding close contact with someone who is infected or at risk* (RR 0.38, 95% CI 0.15–0.97) and a *wearing mask* (RR 0.14, 95% CI 0.04–0.53) were associated, this is compatible with the also significant result of the variable *contact with a suspected or confirmed cases of COVID-19*.

We tested the feasibility of a twice a month RT-PCR testing strategy. Considering a prevalence of 0.07% and accuracy of 0.05) 1,258 participants should be tested to find one positive.

## Discussion

In Switzerland, the “Ciao Corona” study, conducted in June/July 2020, October/November 2020, and March/April 2021 with 2,585 children, found 2.8% (95%CI 1.6–4.1%) SARS-CoV-2 IgG, IgM and IgA seroprevalence [26, 27]. In Germany, a study conducted during May and June 2020 founded 0.6% seroprevalence for students and school staff and 0.7% at the follow up, in September/October 2020 [28]. A population study carried out with children under 18 years of age in Catalonia found a lower seroprevalence than what we found in our study, of 4.4% between March and April 2020. This difference may reflect the difficulty of diagnosing asymptomatic youngers especially during the initial period of the pandemic, emphasizing the importance of active surveillance of school sentinel populations, for the timely detection of respiratory viruses [29].

As expected, in our study there was a significant increase in SARS-CoV-2 seroprevalence, in the school staff group, which can be explained by the increase of vaccination coverage. According to PADRIS data the vaccine coverage in school staff went from 78% and 0.6% in April 2021 to 84.3% and 35.6% in June 202, partly and fully vaccinated respectively. At the time of seroprevalence data collection in this study, vaccines were not approved for people under 18 years.

The prevalence of active SARS-CoV-2 infections detected by the project was low considering the overall prevalence and incidence from Catalonia during the same period [3, 20, 30, 31]. This suggests that public health strategies such as testing of symptomatic individuals and contact tracing efforts were effective at identifying an active infection at school, even the asymptomatic population [28]. Another study proceeded during in December 2020, in a high community transmission period in Switzerland [17] found, a positive PCR in none of the teacher and one child and Antigen positive test in 7 (1.1%) children and 2 (3.0%).

Considering detected and self-reported infections in our longitudinal study we found a low incidence of COVID-19 infections, consistent with other studies that have very similar results to ours [28, 32–34]. Also, there are studies suggesting that higher community incidence, diagnostic issues [30], demographic and economic aspects are determinants in the variation of different rates detected, as showed in these studies [16, 35, 36].

The association between socioeconomic status and SARS-CoV-2 infection was different depending on the period of data collection. First, at the beginning of the pandemic, improved economic situation was positively associated with having been infected. This could be explained by the fact that the most affected population were those who worked and travelled than those who were respecting the lockdown measures. Then, during the follow-up we observed a new trend where a higher infection risk was associated with lower economic status. This provides important clues to understanding the COVID-19 burden in different economic and demographic contexts [37]. Population-based studies found similar results where heterogeneity in incidence and mortality rates [32, 35, 36] were associated with socioeconomic status showing the importance of planning sanitary policies oriented to the territorial characteristics and specific inequities [38, 39], such as in a follow-up study in Brazil that found a high incidence in children living in a slum area [16].

At baseline, contact with suspected or confirmed cases, especially at home, was positively associated with SARS-CoV-2 infection, as observed in a study that found that physical distancing measures, including limited close contacts while school remained open, controlled SARS-CoV-2 transmission [40]. However, school contacts had a negative association with this outcome, showing how well-implemented sanitary protocols make the safe opening of schools possible, consistent with other studies that found an association between low transmission and, sanitary recommendations and preventive measures [6, 41].

In the longitudinal component analysis, all contact patterns were a risk factor, especially when contact was at home, consistent with previous studies that demonstrated an increased risk of infection associated with household contacts in Catalonia [15, 31, 42] and modeling studies that demonstrated an increased risk for infection in household contacts [43, 44]. Even contact tracing studies found no typical or frequent child-adult transmission [16, 45, 46] and a low contribution of children in the secondary cases [47, 48], which showed that children do not seem to be the main source of infection [11, 49].

Interestingly, contact with healthcare professionals was not associated with the infections in our study. Our hypothesis to explain that is the compliance of preventive measures at home when health care workers were exposed to risk situations, however, we need more studies to understand the role of HCW in this transmission model.

Perceived knowledge was positively associated with infection, which may indicate either knowledge acquired due to a previous infection, or the large amount of lay knowledge consumed by the young and indeed the general population [49, 50].

Knowledge of COVID-19 and risk perception may have been due to the occurrence of a previous infection, which would not explain the occurrence of a later infection. A high level of risk perception of exposure might indicate they understood the risks they had taken. Other studies also show that risk perceptions, behaviors and compliance with sanitary measures are associated with levels of knowledge [51–53].

As with other studies [15, 17, 47] our results reinforce that the transmission by children in the school setting did not appear to make a major contribution to the spread of the virus, especially for the youngest children. This supports the decision of many countries to keep schools open while following several public health measures and safety protocols to control the transmission of the virus. Our study also reinforces the idea that the strategy based on an active sentinel surveillance for detection of acute SARS-CoV-2 infections followed by isolation of bubble groups seems to be more effective in scenarios with susceptible groups and rapid transmission.

Approximately half of the target population agreed to participate and considering the difficult circumstances schools and families were experiencing because of the pandemic; we consider this proportion to be quite acceptable. As a matter of fact, it is similar to other studies where 75% and 25% of students and staff participated respectively [28] or with 49% of child participation [17].

## Limitations

Although the overall participation rate in our study was 45.4%, it was proportionately higher among school staff (72%) than students (41%), this suggests that given the fact that higher sociodemographic heterogeneity (nationality, language, socioeconomic status) was higher among students than staff, some of these factors could have also influenced participation. There were no difficulties in implementing the study and all sentinel schools gave us excellent feedback for the associated activities.

Because of the sample of the schools and the participation rate, the study population may not be representative of all schools in Catalonia. Nevertheless, the heterogeneity of the included school’s information from different socioeconomical scenarios with a big enough study population. As a sentinel population approach, the objective of the CSSNC is not to extrapolate parameters, but to complement formal epidemiological surveillance systems by means of monitoring them steadily over time population studied. The data presented were gathered before the Omicron variant circulation and therefore our findings may not apply during the Spanish sixth wave or other future scenarios related to potential new variants and vaccine recommendations for children.

As a cross-sectional design, association should be interpreted with caution, without attributing causality. Variables such as distal characteristics must be interpreted differently from variables that can change over time such as knowledge, behavior, and contact patterns, which are influenced by the occurrence of the disease. Moreover, the acceptability, compliance, and prevention behaviors, may have been directly affected by the course of the pandemic.

There were some limitations to our longitudinal analysis as the small number of acute infections made impossible to apply a multivariate analysis. Also, with community public health measures occurring simultaneously with the schools’ own protocols it was difficult to evaluate these determinants separately. In addition, there was a poor distribution of confounders between groups, which can also have very different sizes, resulting in a loss of statistical power in a multivariate model.

## Conclusions

This study offers a unique perspective on the prevalence and incidence of SARS-CoV-2 infection among students and school staff in Catalonia, an important result considering the difficulty of detecting the virus among asymptomatic young people, as well as regarding the compliance and effectiveness of public health measures implemented in these schools in the transmission of SARS-CoV-2.

The CSSNC demonstrated, for the first time in Spain, the feasibility of correlating individual socio-epidemiological data and data on the prevalence and incidence of SARS-CoV-2 in the school environment, even during the difficult acute period of the pandemic. Despite the high prevalence and community incidence of SARS-CoV-2 in Catalonia during the study period, this project found a low prevalence and incidence of active infections in the school population, suggesting that the prevention methods adopted by schools, together with other strategies of health care, such as testing and contact tracing, were effective in containing transmission in educational settings.

Monitoring of SARS-CoV-2 biological markers and their behavioral and structural determinants over time in sentinel schools is crucial to assess the situation of the COVID-19 pandemic and provide relevant information to inform guidelines and policies to increase safety among students and staff in school environments. Apart from identifying multilevel transmission determinants for SARS-CoV-2 among students and school staff, they may also be useful to describe the spread of other infectious diseases such as influenza and other respiratory viruses and facilitate healthier learning environments for all.
